# *FTO* Gene Polymorphisms and Their Roles in Acromegaly

**DOI:** 10.3390/ijms241310974

**Published:** 2023-06-30

**Authors:** Aleksandra Jawiarczyk-Przybyłowska, Justyna Kuliczkowska-Płaksej, Katarzyna Kolačkov, Agnieszka Zembska, Jowita Halupczok-Żyła, Małgorzata Rolla, Michał Miner, Marcin Kałużny, Marek Bolanowski

**Affiliations:** Department and Clinic of Endocrinology, Diabetes and Isotope Therapy, Wroclaw Medical University, Wybrzeże Pasteura 4, 50-367 Wrocław, Poland; justyna.kuliczkowska-plaksej@umw.edu.pl (J.K.-P.); jowita.halupczok-zyla@umw.edu.pl (J.H.-Ż.); michal.miner@umw.edu.pl (M.M.);

**Keywords:** acromegaly, *FTO*, polymorphism, cardiovascular diseases

## Abstract

The major causes of both morbidity and mortality in patients with acromegaly are cardiovascular diseases (CVDs). The polymorphisms of the fat mass and obesity-associated gene (*FTO)* are associated with obesity, as well as with an increased risk of CVDs. The aim of the study was to determine the relationship of risk alleles of four *FTO* gene polymorphisms with selected parameters of lipid and glucose metabolism as well as with IGF-1 and GH levels in the group of patients with acromegaly compared to the control group. The study group consisted of 104 patients with acromegaly and 64 healthy subjects constituting the control group. In the whole acromegaly group, the data reveal that the homozygous for risk allele carriers (rs1421085, rs9930506, rs9939609) as well as carriers of only one risk allele have lower IGF-1 concentrations. In the well-controlled acromegaly group, the homozygous for three risk allele carriers of *FTO* gene polymorphisms have lower HDL cholesterol concentration (rs1121980, rs1421085, rs993609). In the cured acromegaly group, homozygous risk allele carriers rs9930506 tend to have higher levels of total cholesterol and LDL cholesterol. These associations are not observed in the control group. Conclusion: there is an association between *FTO* gene polymorphisms and the metabolism of lipids, suggesting that the *FTO* gene may be associated with higher CVD risk in patients with acromegaly. In addition, there is an association between *FTO* gene polymorphisms and IGF-1, implying that *FTO* gene may influence/modify IGF-1 synthesis. Further investigation on a larger scale is required to provide more precise evidence.

## 1. Introduction

Acromegaly is a rare disease, caused by the overproduction of growth hormone (GH), which, consequently, leads to the hypersecretion of insulin-like growth factor-1 (IGF-1). The main symptoms of acromegaly are typical changes in appearance as well as many systemic complications. Acromegaly is characterised by three- to five-fold higher mortality than in the general population. An increased prevalence of some cardiovascular risk factors such as dyslipidemia, hypertension, obesity, impaired glucose tolerance, or diabetes mellitus secondary to acromegaly is typical for acromegaly [1,2,3,4,5]. The major causes of both morbidity and mortality in patients with acromegaly are cardiovascular diseases (CVDs), respiratory system diseases, and cancer. The latest meta-analyses show that the main cause in the last decade is cancer. It may be associated with a higher life expectancy in the population of patients with acromegaly and a reduction in the frequency of CVDs, which results from more effective treatment of patients [6,7,8,9]. The mortality in acromegaly can be reduced when patients are treated in specialized centres using a multimodal approach. Due to this, not only can a reduction in GH/IGF-1 be achieved but also the control of comorbidities. This results in increased survival of patients as well as in the improvement in their quality of life [9].

Many population-based studies indicate the role of genetic background in CVDs and associated metabolic disturbances. The advancement of molecular biology techniques allowed for the examination of the genetic background of many diseases. The basis of interindividual genetic variability are single nucleotide polymorphisms (SNPs), resulting from point mutations: transitions or transversions. SNPs account for most of all human DNA variability and are defined to occur in at least 1% of the population, which makes them different from the traditional mutation, which occurs much less often or leads to gene dysfunction or has clinical consequences. The value used to determine the frequency of the allele in the population is minor allele frequency (MAF). It means the frequency of the rarer allele. In 2007, within three months, two studies showed the discovery of *FTO* gene as the first genome-wide association studies (GWAS)-identified obesity susceptibility gene [10,11,12]. According to the previous studies, the polymorphisms of *FTO* are associated with obesity as well as with an increased risk of CVDs [13,14,15,16].

The *FTO* gene is a polymorphic gene located on chromosome 16 (16q12.2), consisting of nine exons covering an area of over 400 kb. It encodes a 2-oxoglutarate-dependent nucleic acid demethylase that is involved in DNA repair and fatty acid and glucose metabolism. The *FTO* mRNA is expressed in many tissues, including adipose tissue, but the highest expression is observed in the hypothalamic nuclei, which controls energy balance and food intake. It also emphasizes the important role of the central nervous system in the predisposition to obesity [17,18]. The region of the brain where *FTO* gene expression is visible is also responsible for the regulation of the GH–IGF-1 axis. It has been suggested *FTO* may also mediate the actions of IGF-1. The data reveal that carriers of the risk allele of *FTO* have lower IGF-1 concentrations, which can lead to obesity. It has been suggested that the GH/IGF-1 axis may be the mediator of the relationship between the *FTO* gene and BMI [19].

The aim of our study was to determine the relationship of four *FTO* gene polymorphisms (rs1121980, rs1421085, rs9930506, rs9939609) with selected parameters of lipid and glucose metabolism as well as with IGF-1 and GH levels in the group of patients with acromegaly compared to the control group. This study is a continuation of an earlier study in which we investigated only the allele frequencies of two *FTO* gene polymorphisms: rs9939609 and rs9930506. In the previous study, we did not have a control group and we did not assess the relationship between disease activity and metabolic parameters [20].

## 2. Results

This was a case–control study. The general characteristics of the study group and the control group are presented in Table 1. The control group was sex- and age-matched.

We show that the distribution of the risk alleles of the analyzed polymorphisms do not deviate between acromegaly and the control group (Table 1), although we observe one difference in the distribution of the risk allele (rs9939609_A) between AAG and CAG (*p* = 0.04) (Table 2).

Body composition analysis shows that the average values of body fat mass and body fat percentage are the lowest in the AAG. On the other hand, the mean values of lean body mass are the highest in the AAG and the lowest in the CG. Differences in the mean percentage of body fat are statistically significant among the AAG and CAG and CG, while in the AAG vs. WCA comparison, the difference is on the verge of statistical significance. The difference in the mean lean body mass in the AAG vs. CG, WCA vs. CG, AAG and WCA and CAG vs. CG groups is statistically significant (*p* < 0.037; *p* < 0.006; *p* < 0.001, respectively). The general characteristics in relation to the activity of acromegaly are presented in Table 2.

In the whole acromegaly group, the data reveal that homozygous risk allele carriers (rs1421085 (CC), rs9930506 (GG), rs9939609 (AA)) as well as carriers of only one risk allele, have lower IGF-1 (IGF-1 x ULN) (rs1421085; *p* = 0.041; rs9930506; *p* = 0.056; rs9939609; *p* = 0.029). In addition, we also observe that homozygous risk allele carriers (rs1121980 (TT), rs1421085 (CC), rs9930506 (GG), and rs9939609 (AA)) have lower levels of glycated hemoglobin but the statistically significant results are for the heterozygous for each polymorphism (*p* = 0.018; *p* = 0.014; *p* = 0.026; *p* = 0.027, respectively) (Table 3).

We did not find any association of *FTO* gene polymorphisms with lipid metabolism in the whole acromegalic group, but we found them when we analyzed acromegalic patients according to the activity of the disease. In the well-controlled acromegaly group, we show that homozygous risk allele carriers of three *FTO* gene polymorphisms have lower HDL cholesterol concentration (rs1121980 (TT), rs1421085 (CC); rs993609 (AA)) (Table 2). In this group, we do not observe the association between polymorphism rs9930506 and lipids. In the cured acromegaly group, we observe that homozygous risk allele carriers of rs9930506 (GG) have a tendency to have higher levels of total cholesterol and LDL cholesterol. In the active acromegaly group, we do not observe these associations. It is important to note that we also do not observe these associations in the control group. There is no association of *FTO* gene polymorphisms with anthropometric parameters including BMI and weight in acromegalic patients (Table 4).

We also analyzed whether the presence of one protective allele is significant in terms of cholesterol concentrations. We show that in the group of controlled acromegaly, patients with at least one protective allele of *FTO* gene polymorphisms have significantly higher HDL values (rs1121980; *p* = 0.011; rs1421085; *p* = 0.005, rs9930506; *p* = 0.052, rs993609; *p* = 0.030). In this analysis, we also observe that patients with at least one protective allele of *FTO* gene polymorphisms have higher levels of total cholesterol (rs1121980; *p* = 0.024; rs1421085; *p* = 0.010, rs993609; *p* = 0.047). In the cured group, the presence of at least one protective allele is associated with lower total cholesterol values (rs1121980; *p* = 0.042, rs9930506; *p* = 0.033).

## 3. Discussion

In recent decades, significant progress has been made in the treatment of acromegaly and its complications, primarily in the treatment of diabetes, heart failure, and hypertension. The early detection of malignant tumors and the progress in their treatment are also important. The consequence is a reduction in the severity and number of complications of acromegaly and a decrease in mortality due to acromegaly. In appropriately treated patients with acromegaly, the mortality rate is similar to that of the general population [6]. Therefore, we strive to understand the background of some complications of acromegaly and predispositions to them.

### 3.1. The Role of FTO and Their Polymorphisms

Due to FTO being a dioxygenase, its enzymatic capacity is expected to decline under hypoxic or ischemic conditions such as myocardial ischemia or infarction. It was reported that FTO removed m6A (N6-methyladenosine) methylation and reversed m6Am modification in cells. This indicates that *FTO* plays a role in the stability of mRNA [21,22]. It was also found that *FTO* has reduced expression in failing mammalian hearts and hypoxic cardiomyocytes, thereby increasing m6A in RNA and reducing cardiomyocyte contractile function. The improvements in *FTO* expression in failing mouse hearts attenuate the ischemia-induced increase in m6A and the decrease in cardiac contractile function. What is more, it was revealed that the overexpression of *FTO* in mouse models of myocardial infarction reduced fibrosis and enhanced angiogenesis [21]. Other studies show that the loss of endothelial *FTO* prevents obesity-induced vascular dysfunction. Krüger et al. demonstrated that the loss of endothelial *FTO* protected patients from obesity-induced insulin resistance, hyperglycemia, and hypertension in the presence of adipose tissue inflammation and obesity. They revealed that endothelial *FTO* is an important regulator in obesity-induced metabolic alterations, which is independent of its known effect on obesity [23].

On the other side, it cannot be ignored that *FTO* is initially found as an obesity-related gene and, as a demethylase, plays a role in promoting adipogenesis. These results indicate that the role of *FTO* in CVDs is different, in different cell types, different tissues, and different pathological conditions. To this moment, sequence variants of *FTO* were observed in obesity in many populations such as European, East Asian, African, Arab, and Brazilian. In the literature, there have also been some contradictions about these dependencies on the individual gene polymorphisms, mainly due to variability, which could be the results of different frequencies of MAF in the selected ethnic groups. What is more, some studies show the influences of variants of *FTO* on the risk of cardiovascular diseases [10,11,12,13,14,15,20,24,25,26,27,28].

### 3.2. Acromegaly and Polymorphisms of Different Genes

In our study, in the well-controlled acromegaly group (WCA), homozygous for risk allele carriers of three *FTO* gene polymorphisms have lower HDL cholesterol concentration (rs1121980, rs1421085, rs993609). In the WCA group, this association for polymorphism rs9930506 is not observed, but in the cured acromegaly group (CAG), homozygous risk alleles carriers rs9930506 tend to higher levels of total cholesterol and LDL cholesterol. What is more, the occurrence of only one protective allele relates to higher levels of HDL. At this time, the only study in which the role of *FTO* gene is assessed in relation to CVD-associated parameters in acromegaly is the study from our centre [20].

Some other research indicates an impact of other polymorphisms of other genes on the course of acromegaly, which could have an impact on the development of early atherosclerosis, determine higher plasma insulin concentration, higher BMI, hypertension, or increased risk of osteoporosis [29,30,31,32,33,34].

Turget et al. showed a significant association between *leptin receptor gene* (LEPR) polymorphism and carotid intima media thickness, which may result in a higher risk of the development of early atherosclerosis in acromegaly. They revealed that *LEPR 223* GG genotype carriers may be at a higher risk of the development of early atherosclerosis in acromegaly than other genotype carriers [29]. Other studies showed that *d3-growth hormone receptor (d3-GHR)* polymorphisms may influence BMI, insulin concentration, and increased systolic blood pressure [30,32]. On the other hand, Franck et al., in their meta-analysis of the influence of *d3-GHR* polymorphism on the courses of acromegaly, showed no effect of this polymorphism on biochemical disease control in acromegaly. This polymorphism also does not affect the response to pegvisomant treatment or dose prediction [35]. Ilhan et al. revealed that *vitamin D receptor (VDR)* polymorphisms may influence the risk of the development of acromegaly as well as its course. They showed the *VDR* FokI ff genotype was associated with a decreased risk, while FokI Ff genotype was associated with a significantly increased risk of acromegaly. What is more, IGF1 levels after the treatment were significantly higher in patients carrying the Ff genotype compared to those carrying the ff genotype. However, they did not find any significant difference across FokI genotypes when the IGF levels after treatment were adjusted according to the upper limit of normal IGF1 [31].

In our study, we did not find an association between the risk of acromegaly and the frequencies of these four *VDR* polymorphisms. However, we reveal a negative correlation between fasting GH and the FokI ff genotype in all acromegaly groups and with IGF-1 levels in the cured disease group. In addition, in the controlled acromegaly group, a positive correlation between Ff genotypes and IGF-1, as well as with IGF-1 according to the upper limit of normal, is revealed. We also show that tt (TaqI), aa (ApaI), and bb (BsmI) genotypes of the *VDR* gene may be associated with better bone quality and microarchitecture (higher TBS). The consequence of this may lead to a lower risk of osteoporotic fractures in acromegaly patients. These findings may suggest a possible role of these polymorphisms during acromegaly [33]. In other research, Oguz et al. indicated that *intercellular adhesion molecule-1 (ICAM 1)* E469K gene polymorphism may be associated with hypertension, higher fasting plasma glucose, and lower HDL-C in acromegaly [34]. These results prompted further research on the role of *FTO* in cardiovascular complications in patients with acromegaly.

### 3.3. FTO Polymorphisms and Their Influence on Lipids Metabolism

According to some previous studies, the presence of the *FTO* risk allele leads to a decreased HDL concentration. In 2008, Freathy et al. reported such an association in a large group of Europeans (more than 17,000) [24]. These associations were also revealed in studies on patients with abnormal glucose metabolism [27,28]. Doney et al. showed that patients with diabetes type 2 and carriers of the allele A of rs9939609 had atherogenic lipid profiles (low level of HDL-C, higher triglycerides) and higher atherogenic index of plasma and BMI. Consequently, these are associated with an increase in the risk of myocardial infarction and cardiovascular death [28]. Other studies revealed that homozygous risk allele carriers of *FTO* rs 9939609 had higher levels of leptin and lower levels of HDL cholesterol in overweight subjects. The difference between carriers TT and AT was also statistically significant, but only for HDL-C. It is more important to note that this association did not change after adjustment for calorie intake, physical activity, and BMI, which may suggest a strong relation between *FTO* and HDL-C [36]. In the Finnish Diabetes Prevention Study (DPS), the subjects with impaired glucose tolerance were genotyped for rs9939609. The research showed that homozygous risk allele (A) carriers of rs9939609 had lower HDL-C in men and, consequently, higher risk of CVDs in men. This effect remained significant, after adjustment factors such as BMI, smoking, lipid profile, hemoglobin glycated concentrations, or treatment. It should be emphasized that this research showed that the effect of the risk allele on cardiovascular mortality was abolished in patients with statin treatment [27].

In our previous study [20], we investigated the frequencies of risk alleles of two *FTO* gene polymorphisms:,rs9939609 and rs9930506, in patients with acromegaly and examined the association with BMI and selected metabolic parameters such as plasma glucose, serum triglyceride, HDL, LDL, and total cholesterol. We demonstrated that risk alleles carriers were associated with decreased HDL concentration and this finding was compatible with previous research [23,26,27,35]. The statistically significant differences between HDL-C concentrations among carriers of different genotypes referred to both analyzed single nucleotide polymorphisms. Homozygous risk allele carriers of rs9939609 had a 1.25-fold lower HDL cholesterol concentration than carriers of the TT genotype. The estimated average decrease in HDL cholesterol concentration per risk allele for rs9930506 was 11.2% [20].

In this study, we show that in the controlled acromegaly group, homozygous risk allele carriers of three *FTO* polymorphisms (rs1121980, rs1421085, rs993609) have lower HDL cholesterol concentrations. We do not observe it for risk allele carriers of rs9930506, but in the cured acromegaly group, we observe that homozygous risk allele carriers of rs9930506 have the tendency to have higher levels of total cholesterol and LDL cholesterol. We do not observe these associations in the whole acromegaly group or in the active acromegaly group. In the active acromegaly group, we do not have patients with homozygous risk allele of three *FTO* gene polymorphisms (rs1421085, (CC); rs993609, (AA); rs-9930506 (GG)). For rs1121980, (TT), we have only one patient who is homozygous. The active acromegaly group is small; in this group, we have only 16 patients. This could be the reason why we do not see differences in metabolic parameters for the group with active acromegaly compared to the other groups. The absence of a risk allele in the active acromegaly group may also affect the observations in the whole acromegaly group. It is more important to note that we do not observe these associations in patients in the control group.

The pathomechanism of the association between the *FTO* polymorphism and HDL concentration is not known and requires further research. Nevertheless, the knowledge about its impact on cardiovascular risk in patients with acromegaly could contribute to other therapeutic decisions in terms of primary prevention. The lowered HDL-C concentration is positively correlated with CVDs risk. Genetic factors are responsible for about 50% of the variability of HDL-C concentrations. Considering the currently available research results, *FTO gene* variants may be also a genetic background of HDL-C concentrations variability.

In our study, we also observe the difference in the lean body mass among groups regarding the activity of the acromegaly and in comparison to the control group. The polymorphisms of *FTO* gene do not affect these results. The obtained results are consistent with previous observations on the influence of GH on body composition in patients with active acromegaly. One characteristic is the reduction in body fat, mainly through the loss of visceral fat. Active acromegaly is also characterised by increased lean body mass and increased accumulation of intramuscular fat. Even though in active acromegaly there is a loss of adipose tissue, metabolic disorders intensify. This is due to the high concentration of GH and the dysfunction of the visceral adipose tissue, which leads to insulin resistance and impaired glucose tolerance. Treatment of acromegaly leads to an increase in body fat and a decrease in lean body mass within a few months of GH normalization [37,38]

It is known that in patients with acromegaly, the inhibition of the activity of lipoprotein lipase and lecithin–cholesterol acyltransferase under the influence of GH and insulin resistance results in an increase in triglycerides and a decrease in the HDL fraction [39]. IGF-1 enhances the uptake of free fatty acids by adipose tissue and the liver and induces lipogenesis. However, the effect of IGF-1 on lipid metabolism seems to be less significant than that of GH [7]. Numerous studies prove that effective surgical treatment, as well as achieving control of the disease with the use of somatostatin analogues, normalize lipid profile parameters (decrease in triglycerides and LDL, increase in HDL) and reduce cardiovascular risk. Effective treatment of acromegaly leads to the inhibition of GH secretion, reduces insulin resistance, and, thus, improves the lipid profile [40,41].

On the other hand, not all patients, despite effective treatment, achieve complete normalization of metabolic parameters. Hence, there is an attempt to find factors that could allow to select those patients in whom, despite effective treatment of acromegaly, there is still no full metabolic improvement. The presented study results show that the occurrence of the rarer allele of individual *FTO* polymorphisms is associated with a lower HDL concentration in relation to carriers of at least one protective allele. The above observations suggest that the presence of risk alleles for *FTO* polymorphism may be considered as a risk factor for metabolic disorders in acromegaly. On the other hand, more research is needed on larger groups of patients to obtain stronger evidence.

### 3.4. FTO Polymorphisms and Their Connection with IGF-1

In the presented study, we also reveal that homozygous of all analyzed risk alleles have a lower level of IGF-1 x ULN. As this time, the association between IGF-1 and *FTO* has been assessed in only one study. Rosskopf et al. showed that in subjects younger than 55 years of age, homozygous risk allele (allele A) carriers of the *FTO* rs992628 exhibited lower serum IGF-I levels adjusted for 5 year age groups, gender, and IGF-I = binding protein 3 levels. Moreover, the *FTO* AA genotype effect on BMI was reduced by including IGF-I. No relationship between *FTO* and IGF-I levels was found in subjects aged 55 years or older [19]. Gao and al. revealed that complete depletion of *FTO* in mice resulted in postnatal growth retardation. In these mice, decreased IGF-1 levels and a significant decrease in bone mineral density in both the whole body and neural *FTO* knockout models were also observed. They argue that it may suggest the function of *FTO* in the hypothalamus–pituitary axis and that the *FTO* mutant mice suffer some degrees of GH deficiency [42].

From this point of view, the knowledge of whether *FTO* regulates GH and/or other hormones secreted by the hypothalamic–pituitary axis could help in the explanation of the physiological functions of *FTO* in the future. Consequently, it might suggest that different variants of *FTO* genes could influence the synthesis and secretion of IGF-1 and, thus, have an effect on treatment effectiveness. This observation needs further research.

### 3.5. FTO Polymorphisms and Their Influence on Glucose Metabolism and Obesity

We also reveal that homozygous risk allele carriers of all analyzed polymorphisms have lower levels of glycated hemoglobin, but these results are not statistically significant. The statistically significant lowest levels of glycated hemoglobin are observed in the heterozygous of all investigated *FTO* polymorphisms. The effect of *FTO* on diabetes risk is still unclear. Firstly, Frayling et al. revealed a 1.15-fold increase in the risk of developing diabetes associated with the presence of the A allele of rs9939609, but further analysis showed that the dependence is a consequence of an increased risk of obesity [12]. Several other studies also pointed out that the influence on the risk of the development of diabetes is secondary to the impact on body mass index or they did not confirm the impact of *FTO* polymorphisms on the risk of diabetes [24,43,44].

On the other hand, several studies reported that the association between the *FTO* locus and the risk of type 2 diabetes remained significant after adjustment for BMI [45,46,47,48]. In the French MONICA study, the role of the A allele of the *FTO* rs9939609 polymorphism on the risk of type 2 diabetes was confirmed, independently of BMI and obesity [45]. In a Scandinavian population study, 2686 people without diabetes were observed and the authors found that 3153 people developed diabetes over 10 years. In their study, the *FTO* polymorphism favored the manifestation of the disease in a significant way after also taking into account the impact on BMI (OR 1.12, *p* = 1.1 × 10^–4^). It was also shown that the effect of *FTO* was independent of the characteristics of central obesity and waist circumference [46]. In a study in a Latvian population (2067 Latvians), rs11642015, rs62048402, and rs9939609 polymorphisms were found to be associated with diabetes, which remained significant after adjusting for BMI. Interestingly, the polymorphism rs57103849 (located in the fourth *FTO* intron) was associated with the early disclosure of diabetes, independent of BMI, and the mechanism of development of diabetes may be different than those manifested by SNPs in the first intron of the *FTO* gene [49]. Ng et al. also show that *FTO* polymorphisms are associated with an increased risk of obesity and type 2 diabetes, with effect sizes similar in East and South Asians and similar to those observed in Europeans. This dependence was independent of BMI [48].

In our analysis, we observe lower concentrations of glycated hemoglobin in acromegalic patients with allele risk. As we know, glucose metabolism disturbances in acromegaly are secondary to higher levels of GH and IGF-1. Acromegalic patients with risk alleles have lower levels of IGF-1, and, maybe for these reasons, we observe lower levels of HbA1c in these patients.

### 3.6. Limitations

The limitation of the presented study is the relatively small sample group. Previous studies underline the importance of using adequately powered studies to assess the effect of a known gene variant on the risk of metabolic complications (diabetes, hyperlipidemia) and, consequently, on secondary characteristics associated with these conditions. On the other hand, acromegaly is a rare disease, so it is very difficult to collect a sufficiently large study group.

## 4. Material and Methods

The study group consists of 104 patients with acromegaly (acromegaly group AG, 64 female, 40 male) and 64 healthy subjects (40 female, 24 male) constituting the control group (CG). All subjects signed the informed consent for participation in this research and the project was accepted by the Bioethics Committee of Wroclaw Medical University. The acromegaly group was divided into three groups according to the activity of the acromegaly: active acromegaly group (AAG, acromegaly de novo or with unsuccessful treatment, 16 patients), well-controlled acromegaly group (WCA, patients during treatment with somatostatin analogues, 56 patients), and successfully operated patients (CAG cured acromegaly group, 32 patients). A total of 10 patients with de novo acromegaly were qualified to the AAG group as well as 6 patients during pharmacological treatment, which did not result in the normalization of IGF-1 and GH. The WCA group included patients with normal IGF-1 levels while on long-acting octreotide (LAR) 20–40 mg intramuscularly every 28 days or long-acting lanreotide 120 mg subcutaneously every 28 or 42 days or pasireotide 40–60 mg intramuscularly every 28 days. The CAG group included patients who experienced successful pituitary adenoma surgery.

The whole blood samples from all patients were collected and stored at −70 °C for further testing. Genomic DNA was isolated from peripheral blood leukocytes according to the protocol of commercial DNA isolation kit (QIAamp DNA Mini Kit, Qiagen GmbH, Hilden, Germany). Simultaneous identification of four *FTO* gene polymorphisms (SNPs) was performed by polymerase chain reaction (PCR) using HotStarTaq Master Mix Kit (Qiagen, Hilden, Germany) and mini-sequencing method (SNaP) according to the protocol of ABI PRISM^®^ SNaPshot™ Multiplex Kit (Thermo Fisher Scientific, Waltham, MA, USA). Amplification was performed using TPersonal Thermocycler (Biometra GmbH, Göttingen, Germany) in the presence of designed pair of primers (synthesized by Generic Biotech, s.r.o. Hradec Králové, Czech Republic); see Table 5 and Table 6.

Products of the reaction were separated by capillary electrophoresis in a DNA sequencer ABI PRISM^®^ 3100 Genetic Analyzer (Thermo Fisher Scientific, Waltham, MA, USA) and analyzed by GeneMapper^®^ Software Version 4.0 (Thermo Fisher Scientific, Waltham, MA, USA).

Descriptive statistics were presented as mean, median, standard deviation, and interquartile range, or counts and percentages. Normality of data was assessed using Shapiro–Wilk test. Analysis of the differences in quantitative parameters between control and the whole acromegaly group as well as comparison of genotypes with a difference of two such as a risk allele were performed using Mann–Whitney test. For activity of acromegaly and comparison of genotypes among three groups, Kruskal–Wallis test with post-hoc analysis using Holm correction was performed. Qualitative comparisons were performed using Fisher’s exact test. Analysis of correlation between quantitative parameters was performed using Spearman correlation coefficient. False-discovery rate correction was applied to control type I error. All calculations were made using the R package for Windows (version 4.2, R Core Team 2021). Statistical results were considered significant when the *p*-value was below 0.05 [50]. 

## 5. Conclusions

In conclusion, the results of our study confirm the association between *FTO* gene polymorphisms and the metabolism of lipids in acromegaly patients and these are consistent with previous studies that have been conducted on different patients populations. It may suggest that *FTO* gene polymorphisms may be associated with higher CVDs risk in patients with acromegaly. It is important to underline that these results also show the association between *FTO* gene polymorphisms and IGF-1, implying *FTO* gene polymorphisms may influence or modify IGF-1 synthesis. Nevertheless, to provide more precise evidence, further investigation on a larger scale is required.

## Figures and Tables

**Table 1:** General characteristic of the groups.

Parameters (Mean±SD)	Acromegaly Group N = 104	Control Group N = 64	*p* Value
Sex	64/104 (61.54% F)	40/64 (62.5% F)	0.99
Age (y.)	58.21 ± 13.4	57.47 ± 11.92	0.58
BMI (kg/m^2^)	29.24 ± 4.9	26.47 ± 3.57	0.001 *
Weight (kg)	83.52 ± 16.56	74.54 ± 14.08	<0.001 *
Lean body mass (kg)	56.07 ± 13.23	48.8 ± 10.45	<0.001 *
GH (ng/mL)	3.28 ± 10.11	0.77± 1.03	<0.001 *
IGF-1 (ng/mL)	206.45 ± 148.85	111.22 ± 32.75	<0.001 *
Glucose (mg/dl)	100.3 ± 21.35	90.55 ± 12.95	<0.001 *
HbA1c%	6.37 ± 1.14	5.79 ± 0.77	<0.001 *
Total cholesterol (mg/dl)	204.28 ± 41.88	191.28±45.93	<0.001 *
LDL (mg/dl)	123.75±36.08	117.4±39.37	0.26
HDL (mg/dl)	56.97 ± 14.39	53.00±12.21	0.075
Trigliceride (mg/dl)	116.98±53.06	111.09±41.2	0.75
Prediabetes	42.67%	12.5%	<0.001 *
Hypertension	58.65%	15.62%	<0.001 *
Rs1121980_T (TT)	18.75%	23.23%	0.40
Rs1421085_C (CC)	17.19%	19.19%	0.45
Rs9930506_G (GG)	20.31%	21.21%	0.5
Rs9939609_A (AA)	17.19%	17.17%	0.39

* *p* values statistically significant < 0.05.

**Table 2:** General characteristics in relation to the activity of acromegaly.

	CG	AAG	WCA	CAG	*p*
BMI	26.47±3.57	29.39±5.58	29.32±4.14 *	29.19±5.85	0.002
Body mass (kg)	74.54±14.08	82.33±18.06	84.32±15.51 *	81.83±17.88	0.002
Lean body mass (kg)	48.86±10.45	59.92±14.12 *	57.15±13.56 *	52.49±11.81	0.002
Body fat (%)	34.69±6.89	27.58±6.96 *	32.95±7.34	35.14±8.32 ^#^	0.015
IGF-1 (ng/mL)	111.32±32.76	492.88±180.14 *	159.35±55.06 *^,#^	145.65±48.69 *^,#^	<0.001
GH (nadir)	0.11±0	13.15±17.93 *	0.43±0.25 *^,#^	0.32±0.31 *^,#^	<0.001
Glucose (mg/dl)	90.55±12.95	104.44±16.77 *	103.89±22.87*	91.94±18.62 ^#,$^	<0.001
Insulin (U/l)	8.31±5.34	12.15±5.96	6.55±4.68 ^#^	6.82±4.7 ^#^	0.003
HbA1C (%)	5.79±0.77	6.53±0.83 *	6.53±1.18* ^,#^	5.96±1.12 ^$^	<0.001
HDL (mg/dl)	56.97±14.39	46.62±10.62 *	55.18±11.77 *	52.38±12.85	0.022
Trigliceride (mg/dl)	116.98±53.06	138.94±43.23	107.85±35.69	102.66±44.54 ^#^	0.032
API	0.29±0.26	0.47±0.19 *	0.28±0.19	0.27±0.26 ^#^	0.019
Prediabetes	12.5%, (n = 7)	58.33% *, (n = 7)	51.44% *, (n = 18)	25%, (n = 7)	<0.001
Diabetes	15.62%, (n = 10)	25%, (n = 4)	37.5% *, (n = 37.5)	12.5% ^$^, (n = 4)	0.016
Dyslipidemia	12.7%, (n = 8)	25%, (n = 4)	1.79% *^,#^, (n = 1)	21.88% ^$^, (n = 7)	0.002
Rs1121980_T (TT)	18.75%	7.69%	23.21%	30%	0.16
Rs1421085_C (CC)	17.19%	0%	19.64%	26.67%	0.18
Rs9930506_G (GG)	20.31%	0%	19.64%	33.3%	0.11
Rs9939609_A (AA)	17.19%	0%	16.06%	26.67% ^#^	0.04

*p* values statistically significant < 0.05; * vs. control group (CG), ^#^ vs. active acromegaly group (AAG), ^$^ vs. well-controlled acromegaly group (WCA); CAG: cured acromegaly group.

**Table 3:** IGF-1 and HbA1c (%) levels in the whole acromegaly group relative to analyzed genotypes.

Polymorphism	Parameter (Mean)	Genotype			*p* Value
Rs1121980		CC	CT	TT	
	IGF-1 (ng/mL)	217±126.86	203.78±157.01	152.7±63.62	0.16
	%ULN IGF-1	0.97±0.58	0.88±0.61	0.69±0.24	0.12
	HbA1c (%)	6.89±1.38	6.04±0.6	6.21±1.26	0.018
Rs1421085		CC	CT	TT	
	IGF-1 (ng/mL)	151.11±53.36	197.14±153.46	221.11±126.93	0.17
	%ULN IGF-1	0.67±0.2	0.86±0.59	0.99±0.58	0.08
	HbA1c (%)	6.27±1.38	6.02±0.6	6.87±1.36	0.014 *
Rs 9939609		AA	AT	TT	
	IGF-1 (ng/mL)	147.42±55.34	189.30±148.07	228.75±130.48	0.059
	%ULN IGF-1	0.66±0.21	0.84±0.58	1.00±0.57	0.041 *
	HbA1c (%)	6.29±1.47	6.04±0.56	6.80±1.36	0.027 *
Rs9930506		AA	AG	GG	
	IGF-1 (ng/mL)	225.22±132.78	194.44±151.39	142.95±52.77	0.050 *
	%ULN IGF-1	0.99±0.58	0.87±0.59	0.65±0.19	0.048 *
	HbA1c (%)	6.84±1.39	6.09±0.55	6.17±1.33	0.026 *

* *p* values statistically significant < 0.05; %ULN IGF-1-percentage of IGF-1 in the upper limit of normal.

**Table 4:** Metabolic parameters in acromegaly groups relative to analyzed genotypes and activity of the disease.

Group	Polymorphism	Parameter	Genotype			*p* Value
Acromegaly group	Rs1121980		CC	CT	TT	
		TCH (mg/dl)	192.32±49.5	195.64±44.9	187.35±45.68	0.85
		HDL (mg/dl)	53.74±13.19	54.38±12.21	51.74±11.0	0.64
		LDL (mg/dl)	114.03±40.80	121.33±39.41	115.00±39.47	0.64
		TG (mg/dl)	122.06±47.00	107.44±38.99	103.13±36.61	0.27
		BMI (kg/m^2^)	30.0±5.25	28.58±5.02	28.75±3.44	0.51
		Body mass (kg)	86.27±17.35	82.20±17.24	82.22±13.79	0.61
	Rs1421085		CC	CT	TT	
		TCH (mg/dl)	182.53±45.09	197.19±45.19	191.94±49.19	0.6
		HDL (mg/dl)	51.53±10.99	54.27±12.24	53.72±12.98	0.65
		LDL (mg/dl)	109.79±38.71	123.29±39.53	113.62±40.21	0.50
		TG (mg/dl)	106.32±39.27	105.35±38.12	122.31±46.26	0.22
		BMI (kg/m^2^)	29.08±3.43	28.66±4.78	29.68±5.49	0.64
		Body mass (kg)	81.58±16.83	83.08±16.83	85.22±18.09	0.76
	Rs9939609		AA	AT	TT	
		TCH (mg/dl)	187.29±44.49	196.28±46.91	190.46±47.39	0.84
		HDL (mg/dl)	52.47±11.26	54.09±12.26	53.40±12.80	0.88
		LDL (mg/dl)	114.12±37.66	122.36±41.12	112.83±38.81	0.68
		TG (mg/dl)	103.94±40.94	106.49±37.36	120.54±46.15	0.28
		BMI (kg/m^2^)	28.92±3.60	28.85±4.60	29.43±5.57	0.86
		Body mass (kg)	79.83±11.76	83.74±16.85	84.91±18.05	0.57
	Rs9930506		AA	AG	GG	
		TCH (mg/dl)	190.30±48.73	194.56±46.23	192.38±44.8	0.93
		HDL (mg/dl)	53.30±13.8	54.09±12.23	52.86±10.92	0.93
		LDL (mg/dl)	112.36±39.66	120.40±40.61	119.71±38.40	0.75
		TG (mg/dl)	122.52±45.64	108.11±38.39	99.19±38.26	0.13
		BMI (kg/m^2^)	29.77±5.45	28.83±4.75	28.48±3.67	0.67
		Body mass (kg)	85.61±17.61	83.85±17.29	79.35±12.36	0.4
AA	Rs1121980		CC	CT	TT	
		TCH (mg/dl)	179.67 ±32.53	206.83 ±67.76	184	0.68
		HDL (mg/dl)	46.83±11.91	50.5±10.89	40	0.39
		LDL (mg/dl)	104.00±	128.5±55.72	123	0.73
		TG (mg/dl)	145.17±43.59	140.67±54.28	104	0.56
AA	Rs1421085		CT	TT		
		TCH (mg/dl)	207.50 ±65.45	179.71±29.7		0.43
		HDL (mg/dl)	48.33±11.57	47.71±11.12		0.83
		LDL (mg/dl)	132.17±54.25	103.57±26.98		0.45
		TG (mg/dl)	136.33±56.3	143.0±40.2		0.73
AA	Rs9939609		AT	TT		
		TCH (mg/dl)	219.25±81.16	180.67±25.79		0.7
		HDL (mg/dl)	51.00±13.74	46.67±9.96		0.54
		LDL (mg/dl)	140.50±67.75	106.22±24.24		0.60
		TG (mg/dl)	141.00±69.59	139.44±37.34		0.83
AA	Rs9930506		AA	AG		
		TCH (mg/dl)	180.75±27.57	211.4 ±72.45		0.71
		HDL (mg/dl)	45.88±0.34	51.40±11.93		0.30
		LDL (mg/dl)	106.8±27.57	132.60±61.27		0.72
		TG (mg/dl)	140.62±39.73	138.80±60.47		0.99
WCA	Rs1121980		CC	CT	TT	
		TCH (mg/dl)	205.95 ±51.31	200.96±41.46	174.08±55.67	0.075
		HDL (mg/dl)	57.68±12.8	56.92±11.45	48.31±8.45	0.037*
		LDL (mg/dl)	124.89±41.18	123.67±37.32	104.38±49.38	0.16
		TG (mg/dl)	115.42 ± 40.05	102.62±33.78	106.85±33.15	0.65
WCA	Rs 1421085		CC	CT	TT	
		TCH (mg/dl)	166.27±51.96	202.19±42.82	205.59±51.31	0.035 *
		HDL (mg/dl)	47.09±7.13	56.77±14.42	57.68±12.8	0.011 *
		LDL (mg/dl)	96.73 ±45.94	125.42±38.45	124.89±41.18	0.06
		TG (mg/dl)	112.18±33.23	100.69±33.18	115.42± 40.05	0.49
WCA	Rs 9939609		AA	AT	TT	
		TCH (mg/dl)	171.67±55.12	197.89±44.65	205.95±51.31	0.12
		HDL (mg/dl)	47.89 ± 7.72	55.82±11.53	57.68±12.8	0.09
		LDL (mg/dl)	102.0±48.06	121.68±40.05	124.89±41.18	0.20
		TG (mg/dl)	109.00±36.26	102.54±32.65	115.42±40.05	0.64
WCA	Rs99305506		AA	AG	GG	
		TCH (mg/dl)	205.94 ± 52.8	196.78±42.65	179.91±57.88	0.22
		HDL (mg/dl)	57.72±13.17	55.93±11.35	49.18±8.95	0.14
		LDL (mg/dl)	124.06±42.21	120.52±38.44	110.09±50.67	0.42
		TG (mg/dl)	119.28±37.4	102.30± 34.32	103.27±35	0.39
CAG	Rs1121980					
		TCH (mg/dl)	161.83 ± 49.24	182.67±41.06	206.89±19.33	0.091
		HDL (mg/dl)	48.17±12.61	51.87±13.65	58.00±12.05	0.31
		LDL (mg/dl)	89.67±41.6	114.73±37.51	129.44±14.21	0.16
		TG (mg/dl)	120.0±69	101.87±36.11	97.67±44.5	0.77
CAG	Rs1421085					
		TCH (mg/dl)	204.88 ±19.63	185.19±40.93	161.83±49.24	0.15
		HDL (mg/dl)	57.62±12.83	52.44±13.38	48.17±12.61	0.41
		LDL (mg/dl)	127.75±14.19	116.50±36.92	89.67±41.6	0.22
		TG (mg/dl)	98.25±47.54	101.31±34.95	120.00±69.06	0.76
CAG	Rs9939609					
		TCH (mg/dl)	204.88 ± 19.63	187.13±41.59	161.00±45	0.11
		HDL (mg/dl)	57.62±12.83	51.67±13.48	50.43±12.97	0.46
		LDL (mg/dl)	127.75±14.19	118.80±37.01	88.57±38.09	0.13
		TG (mg/dl)	98.25±47.54	104.67±33.41	110.14±68.23	0.88
CAG	Rs9930506		AA	AG	GG	
		TCH (mg/dl)	161.00±45.00	183.46±43.41	206.10±18.39	0.065
		HDL (mg/dl)	50.43 ±12.97	51.31±14.24	56.9±11.8	0.43
		LDL (mg/dl)	88.57±38.09	115.46±14.24	130.30±13.67	0.076
		TG (mg/dl)	110.14 ± 68.23	108.38±34.12	94.70±42.99	0.69

AA—active acromegaly, WCA—well-controlled acromegaly, CAG—cured acromegaly group, TCH—total cholesterol, TG—trigliceride, * *p* < 0.05 statistically significant.

**Table 5:** PCR primers.

*FTO* SNP	Forward Primer 5′-3′	Reverse Primer 5′-3′
rs1421085	CTTCCAGGCAAAAGCAGGAG	CAGTGGAGGTCAGCACAGAG
rs1121980	AACAAGGAGACAGCAATGGA	CTCAGTAGATGTGTTAATGA
rs9930506	TGGAGAATGATGAGAATGTA	GCAATTTAAGTAATGCCTAT
rs9939609	CACTAACATCAGTTATGCAT	CCATTTCTGACTGTTACCTA

**Table 6:** SNaP primers.

FTO SNP	Primer 5′-3′
rs1421085	GTAGCAGTTCAGGTCCTAAGGCATGA
rs1121980	CAGGTGGATCTGAAATCTCA
rs9930506	ATCCAATATTAGGGACACAAAAAGGGACATACTAC
rs9939609	TGTCTGAATTATTATTCTAGGTTCCTTGCGACTGCTGTGAATTT

## Data Availability

The original contributions presented in the study are included in the article, further inquiries can be directed to the corresponding authors.
